# On the Validity of the Autobiographical Emotional Memory Task for Emotion Induction

**DOI:** 10.1371/journal.pone.0095837

**Published:** 2014-04-28

**Authors:** Caitlin Mills, Sidney D'Mello

**Affiliations:** University of Notre Dame, Notre Dame, Indiana, United States of America; The University of Queensland, Australia

## Abstract

The Autobiographical Emotional Memory Task (AEMT), which involves recalling and writing about intense emotional experiences, is a widely used method to experimentally induce emotions. The validity of this method depends upon the extent to which it can induce specific desired emotions (intended emotions), while not inducing any other (incidental) emotions at different levels across one (or more) conditions. A review of recent studies that used this method indicated that most studies exclusively monitor post-writing ratings of the intended emotions, without assessing the possibility that the method may have differentially induced other incidental emotions as well. We investigated the extent of this issue by collecting both pre- and post-writing ratings of incidental emotions in addition to the intended emotions. Using methods largely adapted from previous studies, participants were assigned to write about a profound experience of anger or fear (Experiment 1) or happiness or sadness (Experiment 2). In line with previous research, results indicated that intended emotions (anger and fear) were successfully induced in the respective conditions in Experiment 1. However, disgust and sadness were also induced while writing about an angry experience compared to a fearful experience. Similarly, although happiness and sadness were induced in the appropriate conditions, Experiment 2 indicated that writing about a sad experience also induced disgust, fear, and anger, compared to writing about a happy experience. Possible resolutions to avoid the limitations of the AEMT to induce specific discrete emotions are discussed.

## Introduction

### Induction

Quite different from folklore psychology, which maintains that emotions and cognition are separate systems in the brain (i.e., left vs. right brain), scientific research has convincingly shown that emotions interact with and impact a large range of cognitive processes including creativity, decision-making, memory, problem solving, and learning, among others [1]–[8]. The common paradigm to investigate the causal effect of an emotion or mood on some dependent variable (e.g., memory, strategy selection, performance) is to experimentally induce an emotion or mood state and compare the dependent variable of interest in the experimental condition to alternate controls (e.g., another emotion or a neutral condition). The effectiveness of this basic experimental paradigm depends upon appropriate methods to induce specific emotions or mood states. Consequently, an array of emotion induction techniques have been used to experimentally induce emotion, such as writing about emotional memories [9], watching emotional film clips [10]–[13], respiratory feedback [14], and many others [15]–[17] (see [17] for a discussion of common affect elicitation methods). One issue that remains unclear is which emotion induction techniques can be used to reliably induce a specific emotion while not simultaneously inducing additional emotions.

The possibility of inducing specific emotions in laboratory experiments has been in question for many years [17]–[20]. For example, one theory argued that attempts to induce specific emotions are unreliable and may even be the cause of inconsistent findings in the literature [19], [21]. According to this theory, multiple emotions, or more general mood states, are more likely to arise as a result of an emotion induction compared to a specific emotion [21]. However, to date, this type of theoretical assertion has not been a major deterrent for the continued use of emotion induction techniques to make claims about effects of a *specific* emotion.

A small number of studies have attempted to test the theoretical claim that specific emotions cannot be reliably induced (without inducing other emotions as well) by investigating the effectiveness of various emotion induction techniques. These studies investigated if an emotion induction technique was effective by assessing its ability to induce an *intended* (target) emotion after accounting for other *incidental* (non-intended) emotions that may also be affected by the emotion induction.

For example, one set of studies examined the effectiveness of three mood induction techniques (i.e., experimenter verbal attack, the Velten procedure, and threat of electric shock) [22]. Their conclusion supported the notion that specific emotions were not effectively induced. Instead, their research revealed that general mood states were induced, rather than a single emotion, in all three emotion induction techniques (i.e., both *intended* and *incidental* emotions were effectively induced). Conversely, other research has demonstrated an opposite pattern of findings when using film clips to elicit individual emotions [10], [13]. These experiments validated a large set of films that could induce either specific emotions or mixed emotions. Even after including the *incidental* emotions as part of the manipulation checks (e.g., statistical analyses assessing the effectiveness of an emotion induction technique), the *intended* emotions were still considered to be effectively induced using the films. Considering the opposing conclusions drawn from the investigations of these four emotion induction techniques, there is still an open question about the other commonly used techniques in the literature.

One such technique that has not yet been fully investigated is the Autobiographical Emotional Memory Task (AEMT), which employs writing about recalled emotional memories to induce emotions [9]. This task is also referred to as an autobiographical recall procedure [23] or a directed writing task [24]. For this task, participants write about a specific memory involving an *intended* emotion (e.g., fear) in vivid detail, which presumably causes participants to *relive* that event, thereby increasing the likelihood that they will *feel* the emotion. This induction technique is quite popular because it is very easy to implement in both lab and real world settings, takes a relatively short time to complete (<10 minutes), and only requires basic writing supplies.

The AEMT is theoretically grounded in network theories of affect [25]–[27]. These theories suggest that nodes representing knowledge are interconnected with other nodes representing emotional information. The interconnected nodes spread activation to one another. Connections between the knowledge nodes and the emotional nodes are reciprocal, so activation of a knowledge node will excite an emotional node and vice versa. For example, writing about a sad experience, such as a funeral of a loved one, would activate the knowledge nodes representing memories of the incident, which would spread activation to the sadness nodes that are connected to those memories. Over time, these reciprocal waves of activation spreading will increase the intensity of sadness, thereby effectively inducing the emotion.

Some theories of affect might also suggest that the AEMT would not be effective since emotions are unlikely to occur in isolation [18], [28]. Instead, multiple emotion states may occur simultaneously with varying degrees of intensity. For example, emotions that share the same valence (e.g., anger and sadness both have negative valence) might have closely related activation nodes, which may cause these different emotions to be activated at the same time. Importantly, if the latter theoretical explanation is true, the effectiveness of the AEMT as a tool to investigate the effect of *specific* emotions may be compromised.

Like most other emotion manipulation techniques, the effectiveness of the AEMT depends upon two factors: (1) the ability to induce one or more *intended* emotions while (2) not inducing different levels of any other *incidental* emotions across conditions. Some changes in incidental emotions are expected and are not necessarily a threat to the AEMT; however, different levels of incidental emotions between conditions highlight a key concern, such that any effects of intended emotions may no longer be valid since differences in incidental emotions may actually contribute to any observed effect. The main question we will address is whether the AEMT is effective, namely, when incidental emotions are also included in the manipulation check. This question is particularly important because the AEMT is still frequently used [29]–[31] and the common manipulation checks do not account for the potential induction of incidental emotions.

In fact, the two common manipulation checks used in conjunction with the AEMT ignore incidental emotions. The first manipulation check compares the post-writing self-reported level for the intended emotion(s) in the experimental condition(s) to the levels of the intended emotions in a neutral (control) condition [32]–[35]. The second manipulation check compares self-reported emotion levels of the intended emotions across conditions rather than simply comparing to a neutral condition. An example would be comparing ratings of the intended emotion fear in a fearful condition versus ratings of fear in an anger condition. Ideally, manipulation checks would compare the levels of incidental emotions across conditions as well, yet this is quite infrequent. One published study that did test for condition differences in the rates of incidental emotions failed to find any effects [36]. However, it is likely that the small sample size of 13 participants did not have the requisite statistical power to detect an effect, as subsequently confirmed with a post-hoc power analysis.

In order to examine how some of the recent studies have addressed the potential issue of incidental emotions being induced, we conducted a literature review of the last five years of *Emotion*, *Cognition & Emotion*, and *Journal of Personality and Social Psychology*. The journals cover a broad readership and have published a number of studies using the AEMT over the last decade, thereby providing information about the current state of the literature regarding its use. Surprisingly, only three of the 15 articles, listed in Table 1, reported monitoring incidental emotions as part of their manipulation check. Furthermore, the manipulation checks used in two out of the three studies did not adequately address the presence of incidental emotions by comparing them across conditions as we elaborate below.

**Table 1:** List of studies using the autobiographical emotional memory task in the last five years in Emotion, Emotion & Cognition, and Journal of Personality & Social Psychology.

Author (year)	Journal	Intended Emotions Measured	Incidental Emotions Measured
Brinol et al. (2007)*	JPSP	happy, sad	Exp 1: unspecified various emotions; Exp 2: differential scales of excited–relaxed, bored–interested
Fishbach &Labroo (2007)	JPSP	-	-
Goetz et al. (2007)	Emotion	-	-
de Hooge et al. (2007)*	Cognition & Emotion	guilt, shame	regret, disappointment, sadness, fear, anger at self, anger at others, dissatisfaction
Dasgupta et al. (2009)	Emotion	anger, disgust	-
Baumann & DeSteno (2010)	JPSP	happy, anger, disgust, sadness	unspecified feeling descriptors
Griskevicious et al. (2010)	Emotion	enthusiasm, contentment, love, amusement, awe, nurturance	sadness, fear, anger, disgust
Hunsinger et al. (2010)	Emotion	general mood	-
de Hooge et al. (2010)*	Cognition & Emotion	shame	guilt, regret, anger, fear, pride
Tsai & Young (2010)	Cognition & Emotion	anger, fear	-
Baas et al. (2011)	JPSP	-	-
Albaraccin & Hart (2011)	Emotion	anger, happy	-
Siegel & Stefanucci (2011)	Emotion	calm, nervous, anxious, at ease, scared	-
Riener et al. (2011)	Cognition & Emotion	composite valence and arousal from “mood-related adjectives”	-
Young et al. (2011)	Cognition & Emotion	anger, sad	Neutral

*Notes.* Asterisks (*) represent whether or not incidental emotions were compared while using the autobiographical emotional memory task. Blank cells represent that no emotions were measured.

One study that compared incidental emotions across conditions found that shame and guilt (the intended emotions) were higher in the respective conditions [37]. This study also did not find any differences in the rates of several negatively valenced incidental emotions. Although this study appears to allay some of the concerns pertaining to the incidental emotions, recent unpublished data by Myers and Tingley yielded a different pattern of results. Their findings indicated that participants assigned to write about specific negative emotional experiences (i.e., anger, anxiety, and guilt) rated other negative emotions higher as well. For example, participants who wrote about an angry experience not only reported feeling higher levels of hostility, but also reported higher levels of anxiety and guilt as well (as measured by subscales on the PANAS-X; [38]). These unpublished findings align with previous research on other emotion induction techniques showing the induction of multiple emotion states, rather than only one [22].

To summarize, the AEMT is a relatively widely used technique for emotion induction in the affective sciences. The effectiveness of the AEMT, however, is not yet as clearly understood as other previously investigated emotion induction techniques (e.g., threat of electric shock or film clips). Most studies that employ the AEMT technique do not adequately test for condition differences in the rates of incidental emotions; therefore, it is important to establish how incidental emotions are affected while using the AEMT for emotion induction. In particular, out of three studies that tested this relationship, one had very low power [36], while the other two yielded conflicting results [37]. Therefore, a closer examination of this method of emotion induction is warranted and timely.

We conducted two experiments to investigate the effectiveness of the AEMT for inducing specific emotions (anger and fear in Experiment 1; happiness and sadness in Experiment 2). Other than the two methodological changes discussed below, the experimental methods were identical to the procedures followed in the studies cited above (e.g., [36], [39]). The first change involved a more thorough emotion manipulation check that included a set of 14 emotions. These included the four intended emotions (anger, fear, happiness, and sadness), three anger-related emotions (irritated, mad, and frustrated), two fear-related emotions (anxious and nervous), one emotion pertaining to sadness (downhearted), two emotions related to disgust (disgust and repulsed), and two happiness-related emotions (amused and joyful). Second, in addition to measuring post-writing emotion levels, we also measured pre-writing emotions levels. This allowed us to investigate the change in emotions after writing as well as ensure that participants had similar affective profiles prior to writing.

Anger and fear were the intended emotions for Experiment 1, and happiness and sadness were the intended emotions for Experiment 2. For Experiment 1, we hypothesized that we would be able to replicate previous findings that anger would be rated higher for those participants who wrote about *angry* experiences and fear would be higher for those who wrote about *fearful* experiences [36], [40]. Based on theories of affect [18], [28], we predicted that the incidental emotions that share a common valence will also be affected after writing due to shared activation of affective states. Specifically, we predicted the other negatively valenced emotions (disgust and sadness) will also be induced after writing essays recalling angry or fearful memories because of the shared activation with the emotions anger and fear. For example, writing about a tragic accident might induce more than one emotion with negative valence. Increases of these incidental emotions at approximately equivalent rates across the angry and fearful conditions would not be alarming in itself; however, increases at drastically different rates across conditions would threaten the internal validity of the AEMT.

For Experiment 2, we chose to investigate two different emotions that did not share the same valence. Namely, we were interested to assess if the findings in Experiment 1 (using negative emotions) would replicate to a different negative emotion, when compared to a positive emotion. Again, failure to find differences in solely the intended emotions, without inducing incidental emotions, raises some issues pertaining to the internal validity of the AEMT.

## Experiment 1

### Method

#### Ethics Statement

The Institutional Review Boards (IRBs) at the University of Memphis and the University of Notre Dame approved the research protocol. Participants filled out a written electronic informed consent in order to begin the experiment. The consent form indicated that the participant could choose to withdraw from the study without penalty at any time. Furthermore, the consent form also stated that any information gathered is considered confidential and no identifying information would be obtained in the study. Participants were also fully debriefed once the study was completed. The purpose for the study was clearly revealed and participants were given the researchers' contact information in the event of any follow up questions or concerns.

#### Participants

Participants were 85 individuals who volunteered to participate for monetary compensation on Amazon Mechanical Turk™ (AMT). AMT allows individuals to receive monetary compensation for completing Human Intelligence Tasks (HITs) online. Previous research suggests that AMT is a reliable and valid source to collect experimental data [41]–[44]. It also has some advantages with respect to diversity of the participants (age, sex, education level), at least when compared to the typical undergraduate samples used in most research studies.

Participation in the current study was restricted to native English speakers from the U.S. who were 18 years or older. Of the participants, 82.3% self-identified as White, 10.6% as Asian, 1.2% as African American, 2.4% as Hispanic, and 3.5% as “other.” Participants' age ranged from 18 to 72 years, with a mean of 33.9 (*SD* = 12.9 years). Females comprised 59% of the sample. Participants were compensated $0.50 for completing the 15-minute study.

There were 43 participants in the *angry* condition and 42 in the *fearful* condition. Of the 43 participants in the angry condition, 86% identified as White, 9.3% Asian, 2.3% African American, and 2.3% “other.” Females represented 46.5% of participants in the angry condition, with a mean age of 35.7 years. In the fearful condition, 78.6% identified as White, 11.9% as Asian, 4.8% as Hispanic, and 4.8% as “other”. Females represented 71.4% of participants in the fearful condition, with a mean age of 32.2 years.

There was a significant difference in gender between *angry* and *fearful* conditions. We conducted a multivariate analysis of variance and found no significant effect of gender on the post-rating writings and no significant interaction between gender and essay condition for the post-rating writings. Therefore, gender was not included in the subsequent analyses.

#### Design

The experiment had a between-subjects design, with participants randomly assigned to an *angry* or a *fearful* condition.

#### Materials

All materials were administered online using the AMT system. The materials included an informed consent, a demographics survey, and an emotion rating survey. The emotion rating survey included 14 ratings of emotions ordered alphabetically (see Introduction for list of emotions). Each emotion was rated from 1 (not at all) to 8 (more strongly than ever). This rating scale has been used in previous emotion induction studies [45]. Participants completed the emotion rating survey before and after writing about the emotional events (pre- and post- emotion ratings, respectively). An example of the rating scale is presented in the Appendix.

#### Procedure

After providing electronic consent, participants were asked to fill out a brief demographics questionnaire, followed by the pre-writing emotion rating survey. Next, on the basis of random assignment, participants were asked to describe an angry or a fearful event with the following instructions: “*Please describe in detail the one situation that has made you the most angry|fearful you have been in your life, and describe it such that a person reading the description would become angry|afraid just from hearing about the situation.*” These instructions were identical to those that have been used in previous studies [23], [41]. Participants typed their responses in a text box and the content of their responses was stored for offline analysis. There was no specific time limit for writing, although, on average, participants finished the experiment in 11 minutes (*SD* = 8 min). Only some of the previous studies have specified time limits for writing and these have ranged from 4 minutes to 12 minutes [32], [33], [46]–[48]. Participants completed the post-writing emotion survey after completing the writing task, after which they were fully debriefed.

#### Data Treatment

Two judges independently scored the responses to ensure that they did in fact contain relevant emotional content. Interrater reliability was obtained on a randomly selected subset of the responses (20%) prior to coding the entire corpus of responses. Perfect reliability was obtained, so the responses were evenly divided among the two raters. Responses were coded as having (a) no relevance to the intended emotion (i.e., completely unrelated to the intended emotion), (b) some relevance to the intended emotion (i.e., discussion of an emotional experience somewhat related to the intended emotion), or (c) considerable relevance to the intended emotion (i.e., clear discussion related to the intended emotion). None of the responses were coded as having no relevance to the intended emotions, and five were coded as having some relevance to the intended emotion, while the remaining 80 responses were rated as very relevant. Responses rated as having some relevance were included in the analyses because these participants did not clearly fail to follow the directions, which was the only criterion used for removal in previous studies. Hence, the subsequent analyses proceeded with the entire set of 85 participants.

However, as an additional precaution analyses were also repeated using only the responses that were coded as having considerable relevance to the intended emotion and the findings did not change for both Experiments 1 and 2.

#### Grouping Emotions

Except for the intended emotions (anger and fear), which were individually examined, the remaining emotions were aggregated into the following groups. The *sadness emotions* included sadness and downhearted, the *disgust emotions* consisted of disgust and repulsed, and the *happiness emotions* included happiness, amused, and joy. The emotional intensity of each group was computed by averaging the intensities of the individual emotions in the group. This method of grouping emotions has been used in previous studies [24], [36], [39], [46]. Cronbach's α was over 0.7 for all emotion groups, except for the pre-rating of the disgust group (α = .684) and the post-rating for the sadness group (α = .696).

#### Treatment of Outliers

Outliers were removed by standardizing the emotion ratings (i.e., computing z-scores) and eliminating the ratings with z-scores greater than 3.00 or less than −3.00. We chose to use +/−3 standard deviations as the cutoff for outliers because it allowed us to minimize data loss (<1 percent), while removing extreme outliers. A total of three emotion ratings were identified as outliers and removed (2 pre-anger-ratings, and 1 pre-disgust-related rating).

All analyses were also conducted without removing outliers and the same major patterns were replicated for Experiments 1 and 2, so we have some confidence that our choice for outlier removal was adequate.

#### Differences in Length

There was no significant difference in response length as a function of condition, *t*(83) = .812, *p* = .419, *M* = 206 words (*SD* = 192) for the *angry* condition and *M* = 176 (*SD* = 135) words for *fearful* condition.

### Results

We used a repeated measures MANOVA to compare the intended emotions (anger and fear) and incidental emotions (disgust-related, happiness-related, sadness-related) in three pertinent ways: (1) testing for condition difference in pre-writing levels of the emotions to ensure that there were no condition differences prior to writing, (2) comparing post-writing levels of emotions to see if they differed across conditions, and (3) assessing the change in emotion levels from pre-writing to post-writing ratings to investigate if there were meaningful changes after writing. Specifically, rating phase (i.e., pre/post) was the within subject factor, emotion condition was the between subjects factor, (i.e., angry/fearful) and the emotion groups were the dependent variables (five in all). Omnibus tests revealed a significant interaction between essay condition and phase (pre- and post-writing), *F*(7,73) = 10.1, *p*<.001, *η_partial_^2^* = .492. Therefore, we will assess the three key comparisons by investigating specific comparisons involved in this interaction. Descriptive statistics on the pre- and post-writing ratings can be found in Table 2.

**Table 2:** Descriptive statistics for pre- and post-writing ratings and effect sizes for angry vs. fearful post-writing ratings in Experiment 1.

	Pre-Writing Emotions		Post Writing Emotions		
	Angry	Fearful	Angry	Fearful	Angry vs. Fearful
Emotion	*M (SD)*	*M (SD)*	*M (SD)*	*M (SD)*	*d*
Anger	1.55 (.889)	1.41 (.706)	**4.16 (1.79)**	**2.29 (1.69)**	**1.08**
Fearful	1.67 (1.04)	1.88 (1.35)	**2.14 (1.60)**	**3.36 (2.18)**	**−.636**
Happy Emotions	3.42 (1.25)	3.82 (1.67)	2.46 (1.41)	3.37 (1.84)	*−.558*
Sad Emotions	2.63 (1.78)	1.96 (1.40)	**3.58 (1.84)**	**2.55 (1.60)**	**.600**
Disgust Emotions	1.30 (.672)	1.48 (.904)	**3.70 (2.27)**	**1.99 (1.45)**	**.900**

*Notes.* Significant effects bolded. Marginally significant effects italicized.

#### Pre-writing levels of emotion

First, we compared the pre-writing rating levels of the five emotion and emotion groups between the *angry* and *fearful* conditions (Item 1). Pairwise comparisons of the five emotion and emotion groups indicate there were no significant differences in pre-writing rating levels across essay conditions (all *p's*>.05), so participants in both conditions started out with similar affective profiles.

#### Post-writing levels of emotion

We compared the post-writing rating levels of the emotions and emotion-groups to determine if there were any differences across essay types (Item 2). Below we discuss the five planned comparisons that were used to examine differences between the *angry* and *fearful* essays. We chose to be conservative by applying a Bonferroni correction to reduce the chance of a Type 1 error; therefore α was set to .01 (.05/5) for these five comparisons.

#### Intended Emotions

There is the central question of whether the intended emotions were significantly different across the emotion conditions (Item 2). There was a significant effect of condition on anger, *t*(83) = 4.98, *p*<.001 with more anger in the *angry* condition (*M* = 4.16, *SD* = 1.79) than in the *fearful* condition (*M* = 2.29, *SD* = 1.69). There was also more fear (*M* = 3.36, *SD* = 2.18) in the *fearful* condition compared to the *angry* condition (*M* = 2.14, *SD* = 1.60), *t*(83) = −2.94, *p* = .004.

#### Incidental Emotions

There were significant condition differences in the incidental emotions. Pairwise comparisons revealed there was a significantly higher level of the disgust emotions in the *angry* condition (*M* = 3.70, *SD* = 2.27), than in the *fearful* condition (*M* = 1.99, *SD* = 1.45), *t*(83) = 4.14, *p*<.001. Similarly, there was a significantly higher level of the sadness emotions in the *angry* condition (*M* = 3.58, *SD* = 1.84), compared to *fearful* condition (*M* = 2.55, *SD* = 1.60), *t*(83) = 2.76, *p* = .007. Surprisingly, there was also more happiness emotions in the *fearful* condition (*M* = 3.37, *SD* = 1.84) than in the *angry* condition (*M* = 2.46, *SD* = 1.41) at a marginally significant level (α = .01), *t*(83) = −2.57, *p* = .012.

#### Pre- vs. Post-Writing Ratings

In order to ensure that the condition differences observed in the post-ratings represented a significant change from pre-writing to post-writing (Item 3), we investigated the level of change for the emotions that were found to be significantly higher in a respective essay condition using pairwise comparisons from the phase (pre/post)×essay condition (*angry|fearful*) interaction. Specifically, these analyses investigated if the intended and incidental emotions significantly increased after writing. For example, if anger does not increase in the angry condition, the higher levels of anger in this condition may not be attributed to an increase as a result of writing about an angry memory; rather, fearful memories might be simply reducing anger, thereby causing a significant difference between conditions. In fact, the reduction of an intended emotion has been previously found when participants reported less sadness after seeing happy facial expressions and vice versa when viewing sad facial expressions during an emotion induction [20]. These comparisons in this section focused only on the emotions that were previously found to be higher in a respective condition because changes in incidental emotions that were not previously found to differ across conditions would not be a concern for the AEMT for obvious reasons.

In the angry condition, we tested anger, disgust emotions and sadness emotions. In the fearful condition, we tested fear and the happiness emotions. A Bonferroni correction was also applied to this set of five comparisons where α = .01 (.05/5). Anger, disgust, and sadness emotions significantly increased from pre- to post-writing ratings in the *angry* condition, all *p*'s<.001. Fear, on the other hand, significantly increased in the *fearful* condition, *t*(41) = 4.14, *p*<.001.

The decrease in happiness emotions in the *fearful* condition was marginally significant, *t*(41) = 2.31, *p* = .026. This decrease was unexpected because there was significantly more happiness emotions reported in the *fearful* essays compared to the *angry* essays. In order to further investigate this pattern, we performed an additional test comparing the rate of change in happiness emotions in the *angry* condition. This test revealed that there was also a significant decrease in happiness emotions from pre- to post- writing ratings, *t*(42) = 4.33, *p*<.001. This indicates that although post-writing happiness emotion levels were higher in the *fearful* condition compared to the *angry* condition, it was not because writing fearful essays caused a greater increase in happiness emotions. Instead, participants in the *fearful* condition exhibited a lower reduction in happiness emotions (pre to post *d* = .253) compared to participants in the *angry* condition (pre to post *d* = .721).

### Discussion

We conducted a thorough manipulation check to test for unintentional consequences associated with applying the AEMT procedure to experimentally induced anger and fear. The results confirmed some expected patterns, and highlighted some interesting patterns regarding the unintended emotions as well. We expected that the intended emotions would be significantly higher in their respective conditions (i.e., post-writing anger would be higher in the *angry* condition and post-writing fear would be higher in the *fearful* condition). Although the results confirmed this expected pattern, there were also some potentially problematic findings for using the AEMT. In the *angry* condition, two incidental emotions (disgust and sadness emotions) increased significantly after writing about an angry experience and occurred at significantly higher levels compared to the *fearful* condition. On the other hand, happiness emotions decreased at a higher rate in the *angry* condition to the extent that there were higher post-writing levels of the happiness in the *fearful* condition following writing. The fact that these three incidental emotions changed significantly across conditions is not by itself threatening to the effectiveness of the AEMT and is actually somewhat expected given previous research on the occurrence of multiple emotions [21]. However, the significant differences found across the angry and fearful conditions are of concern for the internal validity of the AEMT as an emotion induction technique. For example, any effects attributed to anger vs. fear might now be attributed to some combination of anger, increased disgust, sadness, and lower happiness.

There was the concern that requiring participants to provide pre-writing emotion ratings might influence subsequent emotions during the AEMT. In fact, this is one of the reasons why several of the previous studies have not measured pre-writing emotions [24], [32], [47], [49], [50]. To address this concern, we collected data on an additional sample of 39 participants who skipped the pre-writing emotion survey and only provided post-writing emotion ratings. Emotion judgments of these participants (post-only group) were compared to the 85 participants who provided both pre- and post-writing emotion ratings (pre-post group). A multivariate analysis of variance with the post ratings of the intended emotions and emotion groups as dependent variables and rating type (post-only and pre-post) as the independent variable did not yield a significant model for rating type, *F* (6,117) = .297, *p* = .937, so we have some confidence that the pre-writing ratings did not subsequently influence post-writing ratings.

Taken together, the results of Experiment 1 lead us to have some misgivings about the validity of the AEMT as an effective method to induce emotions, particularly for negative emotions. We used anger and fear as intended emotions and found that disgust emotions and sadness emotions were also induced in the *angry* condition. Previous research has reported mixed results regarding the effectiveness of different emotion induction techniques to induce specific emotions [22], [51], so there was still a question about whether the AEMT can be used to induce specific emotions. This experiment provided initial evidence for doubting the effectiveness of the AEMT, which aligns with some of the previous work using other emotion induction techniques [22]. Since this experiment involved writing about two negative emotions, the next step was to investigate if these effects also occurred when participants were asked to recall and describe emotional experiences that differed in valence. This was accomplished by asking participants to write about *happy* or *sad* experiences in Experiment 2.

Similar to Experiment 1, we predicted that happiness would be higher for those who wrote about *happy* experiences and sadness would be higher for those who wrote about *sad* experiences. Based on findings from Experiment 1, we also hypothesize that we would find differences in the incidental emotions (disgust emotions, anger emotions, and fear emotions) between the two conditions. Specifically, since sadness emotions and disgust emotions were induced in the angry condition in Experiment 1, we predict that anger emotions and disgust emotions will be similarly induced in the sad condition in Experiment 2.

## Experiment 2

### Method

#### Participants

Participants were 96 individuals who volunteered to participate for monetary compensation on AMT. Similar to Experiment 1, participation was restricted to English speakers from the U.S. Each participant was paid $0.50 for the 15-minute study. One participant only wrote four words and was removed for failing to generate enough content to sufficiently complete the task. The average age of participants was 32.2 years (*SD* = 12.0 years) and 59% percent were females. 71.6% were Caucasian, 8.4% were Asian, 5.3% were African American, 6.3% were Hispanic, and 8.4% self-identified as “other”.

There were 50 participants in the *happy* condition and 45 in the *sad* condition. Of the 50 participants in the happy condition, 78% identified as White, 6% Asian, 6% African American, 4% as Hispanic, and 6% “other.” Females represented 62% of participants in the angry condition, with a mean age of 32.4 years. In the sad condition, 64.4% identified as White, 11.1% as Asian, 11.1% as African American, 6.7% as Hispanic, and 6.7% as “other”. Females comprised 55.6% of participants in the fearful condition, with a mean age of 32.2 years. Tests for differences in the three demographic variables (age, ethnicity, and gender) did not reveal any significant differences between the *happy* and *sad* conditions. Thus, these variables were not included in subsequent analyses.

#### Materials and Design

The experiment had a between-subjects design with participants being randomly assigned to a *happy* or a *sad* condition. The materials were identical to Experiment 1, except the instructions asked participants to recall and describe a *happy* or *sad* experience.

#### Procedure

The procedure was identical to Experiment 1.

#### Data Treatment

The same judges from Experiment 1 scored the responses using the same categories to judge relevant emotional content. The responses were divided evenly across judges. None of the participants' responses lacked relevant emotional content, three had some relevant emotional content, and the remaining 92 had considerable emotional content. Hence, all 95 participants were included in the analyses.

#### Treatment of Outliers

Similar to Experiment 1, outliers were removed by standardizing the emotion ratings (i.e., computing z-scores) and eliminating ratings with z-scores greater than 3.00 or less than −3.00. A total of eleven emotion ratings were removed (1 pre-anger rating, 1 pre-fear rating, 2 post fear-related emotion ratings, 2 post-anger group, 4 pre-disgust group ratings, and 1 post-disgust group rating). The pattern of results was not affected by outlier removal.

#### Differences in Length

An independent samples *t*-test revealed that although there was a trend for longer responses (in terms of words) in the *sad* condition (*M* = 200, *SD* = 199) compared to the *happy* condition (*M* = 140, *SD* = 116), the difference was not statistically significant, *t*(109) = 1.83, *p*>.05.

#### Grouping Emotions

While happiness and sadness were analyzed individually, the remaining emotions were grouped as follows: anger, mad, frustrated, and irritated were the anger emotions; fear, anxious, and nervous were the fear emotions; and disgusted and repulsed were the disgust emotions. Cronbach's *α* was above 0.7 for all emotion groups.

### Results

Similar to Experiment 1, we focused on three key comparisons: (1) testing for condition difference in pre-writing levels of the emotions, (2) comparing post-writing levels of emotions across conditions, and (3) assessing the change in emotion levels from pre-writing to post-writing ratings. As in Experiment 1, a repeated measures MANOVA was conducted where rating phase (i.e., pre/post) was the within subject factor, emotion condition was a between subjects factor, (i.e., happy/sad) and the emotions or emotion groups were the dependent variables (happiness, sadness, anger emotions, disgust emotions, fear emotions). Omnibus tests revealed a significant interaction between essay condition and phase (pre- and post-writing), *F*(7,80) = 15.57, *p*<.001, *η_partial_^2^* = .577. Therefore, we addressed the three key comparisons by investigating specific comparisons involved in this interaction. Descriptive statistics on the pre- and post-writing ratings can be found in Table 3.

**Table 3:** Descriptive statistics and effect sizes for pre- and post-writing ratings and effect sizes for happy vs. sad post-writing ratings in Experiment 2.

	Pre Writing Emotions		Post Writing Emotions		
	Happy	Sad	Happy	Sad	Happy vs. Sad
Emotion	*M (SD)*	*M (SD)*	*M (SD)*	*M (SD)*	*d*
Happiness	4.26 (1.79)	4.16 (1.95)	**5.06 (2.05)**	**3.09 (2.10)**	**.952**
Sadness	2.26 (1.81)	2.04 (1.83)	**2.14 (1.70)**	**4.96 (2.15)**	**−1.45**
Anger Emotions	2.04 (1.14)	2.32 (1.64)	**1.79 (1.29)**	**2.83 (1.93)**	**−.636**
Fear Emotions	2.38 (1.46)	2.22 (1.43)	1.76 (1.12)	2.18 (1.37)	−.341
Disgust Emotions	1.40 (.842)	1.32 (.756)	**1.36 (.941)**	**2.46 (1.92)**	**−.727**

*Note.* Significant effects bolded.

#### Pre-Writing Levels of Emotion

We compared the pre-writing rating levels of the five emotion and emotion groups (happiness, sadness, anger emotions, disgust emotions, and fear emotions) between the *happy* and *sad* conditions (Item 1). Pairwise comparisons indicate there were no significant differences in pre-writing rating levels across the *happy* and *sad* essay conditions (*p*>.05). We considered this to be indicative that participants began the writing process with similar affective profiles across conditions.

#### Post-Writing Levels of Emotion

The post-writing rating levels of the emotions and emotion-groups were compared across essay conditions (*happy* vs. *sad*) to determine if there were any differences (Item 2). We conducted five planned comparisons to investigate the differences, which are discussed below. As in Experiment 1, α was set to .01 for these five comparisons by applying a Bonferroni correction (.05/5).

#### Intended Emotions

There was a significantly higher level of happiness in the *happy* condition (*M* = 5.06, *SD* = 2.05) than in the *sad* condition (*M* = 3.09, *SD* = 2.10), *t*(93) = 4.63, *p*<.001. Similarly, there was a significantly higher level of sadness in the *sad* condition (*M* = 4.96, *SD* = 2.15) than in the *happy* condition (*M* = 2.14, *SD* = 1.70), *t*(93) = −7.11, *p*<.001.

#### Incidental Emotions

There was a significantly higher level of the anger emotions in the *sad* condition (*M* = 2.83, *SD* = 1.93) compared to the *happy* condition (*M* = 1.79, *SD* = 1.29), *t*(91) = −3.10, *p* = .003. Similarly, significantly higher levels of the disgust emotions were also reported in the *sad* condition (*M* = 2.46, *SD* = 1.92) compared to the *happy* condition (*M* = 1.36, *SD* = .941), *t*(92) = −3.57, *p* = .001. There were no significant condition differences in the post-writing levels of fear, (*M* = 2.18, *SD* = 1.37 and *M* = 1.76, *SD* = 1.12), *t*(91) = −1.65, *p* = .102.

#### Pre- vs. Post –Writing Ratings

As in Experiment 1, the next step was to ensure that the condition differences observed in the post-ratings reflected a significant change from pre-writing to post-writing (Item 3). To address this, we investigated pairwise comparisons from the phase (pre/post)×essay condition (*happy*/*sad*) interaction. Again, we specifically looked at the level of change for the emotions that were found to be significantly higher in the respective essay conditions. In all, we conducted four tests comparing the change from pre- to post-writing ratings and set an alpha level to address these four comparisons (α = .0125). In the *happy* condition, we only tested if happiness changed from pre- to post- writing. In the *sad* condition, we tested for increases in sadness, anger emotions, and disgust emotions.

Happiness significantly increased in the *happy* essay condition, *t*(44) = 4.22, *p*<.001. All three emotions that were found to be significantly higher in the *sad* condition (sadness, anger emotions, and disgust emotions) were found to significantly increase from pre-writing to post-writing, *p*'s<.01.

### Discussion

Experiment 2 investigated the fidelity of the AEMT to induce happiness and sadness. As expected, the intended emotions were successfully induced (i.e., post-writing happiness was higher in the *happy* condition and sadness was higher in the *sad* condition). Therefore, we have replicated past findings that have used this method to induce happiness and sadness [47], [48], [52]. However, two incidental emotions (anger, disgust) were also induced in the *sad* condition. This replicates a key finding from Experiment 1 that writing about a negative emotional event also induces incidental emotions with similar valence that are categorically different from the intended emotion.

## General Discussion

Emotion induction methods are instrumental for experimental investigations into the effects of emotions. Previous investigations of other emotion induction methods have produced mixed results about the effectiveness of various emotion induction methods when incidental emotions are included in the manipulation checks [10], [13], [22]. Therefore, we conducted two experiments to systematically investigate the effectiveness of the AEMT. This research is informative for the use of AEMT, considering common emotion manipulation checks do not sufficiently ensure that only the intended emotions, but not incidental emotions, are induced at different levels between conditions [37], [40], [53]–[65]. Pre-writing emotion levels were also not considered in these previous studies. To address these concerns, we measured both intended and incidental emotions as well as pre-writing emotion levels. In this section, we take stock of the findings, discuss some limitations with our study, and provide recommendations for those who want to use the AEMT task in the future.

### Major Findings

The results across two experiments indicated that in addition to the intended emotions, certain incidental emotions were also induced as a result of writing about intense emotional events. For Experiment 1, disgust emotions and sadness emotions were induced in the *angry* condition, but not in the *fearful* condition. Therefore, any effect associated with *anger* cannot be attributed to anger alone, but instead to a combination of anger, disgust, and sadness, which all increased from pre- to post- writing. For Experiment 2, anger emotions and disgust emotions were higher in the *sad* condition compared to the *happy* condition and these emotions increased from pre- to post- writing in the *sad* condition. Likewise, any effects cannot be solely attributed to sadness, but instead to a combination of sadness, anger, and disgust. Importantly, this is problematic for a comparison of the effects of two discrete emotions because any differences may actually be due to the incidental emotions, or to a blend of the emotions [21].

The effect sizes for the intended emotions in both experiments were consistent with the medium to large effect sizes obtained in other studies that have used this method to induce emotions [24], [36], [39], [46]. Effect sizes were computed using Cohen's *d*. Based on the recommendation by Cohen (1992), .20 is considered a “small” effect, .50 is considered a “medium” effect, and .80 is considered to be a “large” effect. Therefore, we are confident that we have replicated the basic finding that writing about an event involving a particular emotion can induce that emotion. However, the effect sizes of the incidentally induced emotions are also notable. In particular, compared to writing about a *fearful* experience, writing about an *angry* experience yielded a medium effect for sadness (*d* = .600) and a large effect for disgust (*d* = .900) in Experiment 1. Similarly, Experiment 2 revealed that writing about a *sad* experience yielded large effects for anger (*d* = .636) and disgust (*d* = .727), compared to writing about a *happy* experience.

These results are not surprising from the perspective of network theories of emotion [18], [25]–[27]. Emotions sharing negative valence, such as anger, fear, and disgust, are expected to be closely related in a semantic network of emotional and cognitive nodes. This is because knowledge representations of particular emotional memories might be simultaneously associated with several emotions of similar valence. For example, the accidental death of a pet might have triggered *sadness* because of the loss, *disgust* about the discovery of the carcass, and *anger* at the person responsible for the death. All three emotions are part and parcel of the emotional episode, and nodes representing this memory would be associated with related emotion nodes, thereby strengthening connections among these emotional nodes. Simply put, it is unlikely that there is a one-to-one relationship between a particular knowledge node and a specific emotion. A one-to-many relationship is more the norm than the exception.

This research further addresses the concerns previously expressed regarding the induction of multiple emotions [22]. Using different emotion induction techniques, such as the Velten procedure, experimenter verbal attack, and threat of electric shock, previous research concluded that it is unlikely that single emotions are purely induced in the laboratory [19], [22]. Conversely, film clips, another emotion induction technique, have actually been reported to be successful in inducing discrete emotions [13] (after accounting for incidental emotions). Therefore, this investigation uniquely demonstrates the threat of inducing different incidental emotions across conditions when using the AEMT.

### Limitations and Resolutions

There are some limitations with the present study. One limitation was that we did not include a *neutral* condition in our experiments (e.g., participants would write about neutral topics, such as mundane everyday experiences). A *neutral* condition may have provided a control condition with non-emotional writing that could be compared with the emotion conditions. Although we did not include a *neutral* condition in our experiments, past research has demonstrated that emotions are significantly different when writing about emotional experience compared to writing about neutral experiences [32], [35]. Therefore, we do not consider the lack of a neutral condition to be a major limitation.

A second limitation involves our selection of intended emotions. Experiment 1 contrasted two negative emotions; Experiment 2 contrasted a negative with a positive emotion. Contrasting two positive emotions (e.g., happiness and pride) would be a useful item for future work.

Another potential limitation is the use of AMT to collect data for these experiments. We were unable to control the participants' environmental factors, since they were all recruited online via AMT. Although we cannot be certain that environmental factors did not affect the emotion ratings in any way, we are still confident that our results provide an accurate portrayal of using the AEMT because we consistently replicated the medium to large effects associated with inducing the intended emotions as observed in past laboratory studies that used this method. Replicating these effects, despite having less control over environmental factors, provides all the more evidence to support the robustness of these findings. Furthermore, a recent study [66] that tracked emotions *during* writing of an emotion found a similar distribution of emotions irrespective of whether participants completed the study in the lab (Experiment 1) or online (Experiment 2).

The last limitation of conducting the studies on AMT is that it does not afford collection of physiological and behavioral measures, such as electrodermal activity and facial expressions, which ostensibly can be used as a more objective emotion manipulation check. Replicating the present studies with physiological measures, along with implicit measures of affect, is an important item for future work.

### Recommendations

The AEMT has a number of advantages as an emotion induction tool, both in the lab and in the field, because it has been shown to be effective in inducing intended emotions, is quite short (<10 minutes), does not require any technology (in contrast to other methods such as viewing films or listening to music), and scales up to group administration. Unfortunately, our examination of this method highlighted some critical concerns that might threaten the internal validity of experiments that use the AEMT method without appropriate manipulation checks in place. Our experiments demonstrated that the AEMT introduces some confounds when it comes to inducing specific negative emotions since writing about an intended negative emotion induced other incidental negative emotions at different rates across conditions. Hence, one recommendation is to limit the use of this technique to induce general mood states, such as positive and negative moods, rather than specific emotions.

There will still be situations where specific discrete emotions need to be elicited. In these situations, our recommendation is that researchers first perform a theoretical analysis in order to identify and measure incidental emotions that are likely to occur along with the intended emotions. If it is subsequently discovered that these incidental emotions significantly differ across conditions, then they should be statistically controlled in the analyses. This paper provides some initial evidence regarding which incidental emotions are likely to increase together, so at the very least, these emotions should be measured and addressed in the analyses.

Finally, one may also use alternative emotion induction techniques when available. For example, researchers have made available a library of film clips that effectively induce six discrete emotions in a manner that overcomes some of the limitations of the AEMT [13]. It has also been proposed that using behavioral emotion inductions (e.g., gifts) or naturally occurring moods might prove to be a better way to compare different emotions [18]. These and alternate emotion elicitation methods (see Handbook of Emotion Elicitation and Assessment [17]) can be used in lieu of the AEMT, if needed.
